# Inflammatory Fibroid Polyp of the Cecum can be Treated by Endoscopic Resection

**DOI:** 10.4103/1319-3767.43281

**Published:** 2008-10

**Authors:** Musthafa Chalikandy Peedikayil, Hindi N. Al Hindi, Mohamed Awad Said Rezeig

**Affiliations:** 1 Department of Medicine (MBC 46), Section of gastroenterology, King Faisal Specialist Hospital and Research Center, Riyadh, Saudi Arabia. E-mail: musthafacpdr@gmail.com; 2 Department of Pathology and Laboratory Medicine, Riyadh, Saudi Arabia; 3 Department of Medicine (MBC 46), Section of gastroenterology, King Faisal Specialist Hospital and Research Center, Riyadh, Saudi Arabia

Sir,

An inflammatory fibroid polyp (IFP) is a rare benign, nonneoplastic, polypoid lesion of the gastrointestinal tract. A 66-year-old hypertensive and diabetic woman was referred to us for the evaluation of severe iron deficiency anemia. Clinically she was obese, pale, with stable vital signs, and physical examination revealed no abnormalities. Laboratory data were unremarkable except for a hemoglobin level of 62 g/L; upper endoscopy was normal. 

**Figure 1:**
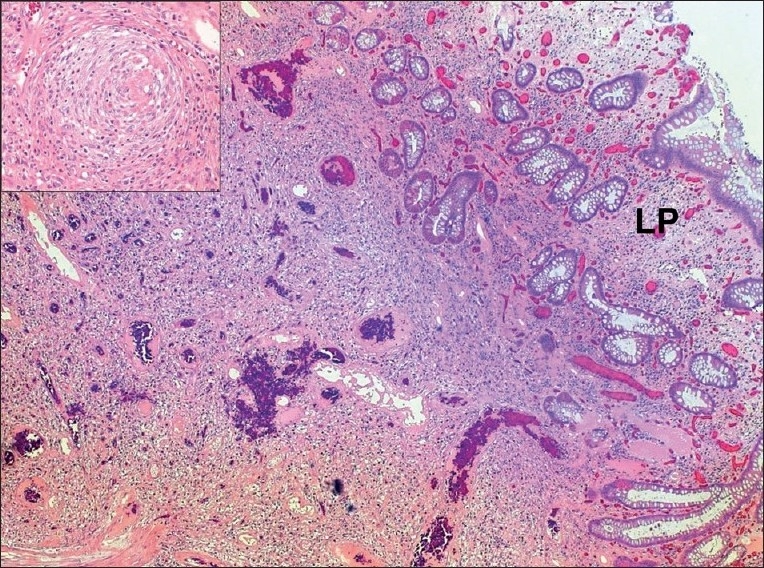
Histological section of the superficial part of the polyp, where the submucosa and part of lamina propria (LP) are replaced by a fibroinflammatory and vascular tissue rich in eosinophils. Many vessels are thick-walled. Inset: high magnification of a vessel with concentric (onion skin-like) fibrosis and eosin stain. Original magnification ×40 (inset ×200)

Colonoscopy revealed a 3-cm sessile, polypoid lesion in the cecum, and biopsy was performed [Figure 1]. A CT scan of the abdomen was normal except for small pericecal lymph nodes. Histology of the polypoidal lesion showed features of an inflammatory fibroid polyp. The patient underwent a second colonoscopy and the polyp was removed using the snare-and-cautery technique without any complications, in small pieces. But at the end the whole polyp was removed leaving only its base.

Microscopically, the lesion was a polypoidal mass of fibromuscular and inflammatory tissue covered by colonic mucosa. The latter was continuous with the underlying tissue and had focal ulceration and granulation tissue formation. The lesion was composed of a highly vascular tissue with bland fibroblast-like cells and an inflammatory infiltrate of lymphocytes, eosinophils, and plasma cells. In some areas, the inflammation was dense with the formation of reactive lymphoid follicles [Figure 1].

Colonic IFP is rare and there have only been a total of 44 cases, including our case, reported in the literature.[1–3] Out of 26 cases of colonic IFP reported by Sakamoto ***et al.***, 17 have been treated surgically. Different techniques have been described for the endoscopic removal of the polyp including the one using the clip-and-cut technique.[4]

In our case, a large polypoid lesion was found in the cecum during colonoscopy done as a part of the diagnostic work-up for iron deficiency anemia. IFPs originate primarily in the mucosa and submucosa, but they can, in rare instances, extend to the muscular layer. In this patient, the IFP was predominantly in the mucosa without any extension to the muscular layer, and hence, we decided to remove this by colonoscopy and the snare polypectomy technique. Follow-up colonoscopies done three months and a year later showed no residual lesions or recurrence. 

As the IFP is a benign polyp and its recurrence is very rare, we believe that its endoscopic removal is an appropriate treatment modality. With wide use of colonoscopy, more such cases of IFP will probably be identified, and awareness of such condition among physicians will help to avoid surgery and resection.
